# Replace, Reprogram, and Relay: A Framework for Glial Cell and Extracellular Vesicle Therapies in CNS Repair

**DOI:** 10.3390/cells15201851

**Published:** 2026-10-12

**Authors:** Mousumi Ghosh, Damien D. Pearse

**Affiliations:** 1 The Miami Project to Cure Paralysis, University of Miami Miller School of Medicine, Miami, FL 33136, USA; dpearse@med.miami.edu; 2 The Department of Neurological Surgery, University of Miami Miller School of Medicine, Miami, FL 33136, USA; 3 The Neuroscience Program, University of Miami Miller School of Medicine, Miami, FL 33136, USA; 4 Biochemistry and Molecular Biology Program, University of Miami Miller School of Medicine, Miami, FL 33136, USA; 5 The Interdisciplinary Stem Cell Institute, University of Miami Miller School of Medicine, Miami, FL 33136, USA

**Keywords:** microglia, extracellular vesicles, glial cell therapy, CNS repair, microglial replacement, astrocytes, oligodendrocyte progenitor cells, remyelination, neurodegeneration, spinal cord injury

## Abstract

**Highlights:**

**What are the main findings?**

- Living glia can provide adaptive, structural, and sustained support functions, whereas glial extracellular vesicles (EVs) deliver molecular cargo packaged before administration through finite exposures.
- Microglial EVs can support repair or propagate inflammatory signals and enhance pathology, depending on producer-cell state and vesicle composition.

**What are the implications of the main findings?**

- The Replace-Reprogram-Relay framework informs therapeutic selection by distinguishing the need to re-place deficient cell populations or functions from modulation of surviving cells, while defining the spatial and temporal delivery requirements.
- Platform selection requires evidence of functional potency, target engagement, and duration of effect. Matched cell:EV comparisons are needed to establish relative benefits, as available studies do not establish that one platform is consistently more effective than the other.

**Abstract:**

Glial cells shape inflammatory resolution, metabolic support, debris clearance, axonal preservation, and myelin repair after CNS injury and neurodegeneration. These functions have motivated two related but non-equivalent therapeutic strategies, either the transplantation and replacement of living glia or the delivery of glia-derived extracellular vesicles (EVs). A viable cell graft can persist, migrate, phagocytose, integrate with host tissue, and adapt its transcriptional and secretory programs after administration. EVs cannot reproduce these autonomous cellular functions, but they can transfer proteins, lipids, metabolites, and nucleic acids as finite, engineerable packages that may be repeated. Microglia provide the principal comparison because the same lineage can be transplanted, replaced, genetically engineered, or used to generate therapeutic EVs. Strategies targeting the microglial compartment include CNS-wide niche repopulation and pathology-responsive engineering, with early human evidence from hematopoietic transplantation in a small uncontrolled cohort with CSF1R-associated microgliopathy, whereas glial EV therapeutics remain predominantly preclinical. Producer-cell state is also critical because microglial EVs can support repair or propagate inflammatory and proteopathic signals. We compare glial cell and EV strategies across CNS trauma and neurodegeneration in terms of biological capability, evidence level, biodistribution, potency, manufacturing, and safety. The proposed framework separates two questions: (1) whether pathology requires replacement of a missing or intrinsically defective cellular population or function (Replace) versus changing the functional state of surviving recipient cells without replacing the population (Reprogram), and, when applicable, (2) what spatial and temporal delivery (Relay) is required, including whether exposure must be local and persistent or distributed and repeatable. Because matched cell-versus-EV studies are rare and the evidence base is asymmetric, this framework is a mechanistic guide for hypothesis generation rather than a ranking of platform efficacy in specific neurological injury or disease paradigms.

## 1. Introduction

Neurological disability is often expressed through neuronal dysfunction or loss, but neuronal function is strongly constrained by the surrounding glial environment. Glia regulate inflammatory resolution, glutamate and ion homeostasis, metabolic support, debris clearance, extracellular matrix remodeling, myelin maintenance, and vascular function. Failure or recovery of these functions can determine whether surviving neural tissue remains dysfunctional or supports repair.

Glial lineages address different components of this environment. Microglia coordinate surveillance, debris clearance, and inflammatory responses. Astrocytes maintain neurotransmitter, ionic, metabolic, extracellular-matrix, and neurovascular homeostasis. Oligodendrocytes provide myelin and metabolic support, and OPCs replenish the oligodendrocyte pool after demyelination. Schwann cells (SCs), a peripheral nervous system glial cell with myelinating capacity, and olfactory ensheathing cells (OEC), a glial cell that ensheathes non-myelinated axons of olfactory sensory neurons from the PNS into the CNS, have also been employed to restore axon function in CNS injury and disease models. These functions are state- and context-dependent and cannot be reduced to the secretion of individual trophic factors.

Therapeutic use of glia spans two biologically distinct product classes. Viable cells can be delivered as focal grafts, used to restore depleted or dysfunctional populations, or engineered to provide an additional or enhanced therapeutic function. EVs harvested from cultured glia carry proteins, lipids, metabolites, messenger RNAs, and noncoding RNAs and can reproduce selected components of paracrine signaling [1,2]. Their acellular nature may facilitate characterization, storage, and repeat dosing, but these properties do not make them equivalent to the parent cell or inherently safe. Nor should EVs be assumed to outperform simpler acellular approaches, as their value must be demonstrated through product-specific delivery, target engagement, and functional potency.

Platform selection begins with the function that the lesion lacks. Loss of a critical or intrinsically defective cell population favors replacement, whereas persistence of competent cells in a maladaptive state creates an opportunity for reprogramming. A separate issue is exposure, as some targets require persistent local activity, whereas others require repeatable delivery across a broad or discontinuous anatomical territory. Microglia are the principal mechanistic model in this review because transplantation, niche replacement, cellular engineering, and EV biology can all be examined within one lineage. Astrocytes, oligodendroglial cells, Schwann cells, and olfactory ensheathing cells are then used to test which principles generalize beyond microglia.

The framework is not an efficacy ranking. Direct cell-versus-EV comparisons under matched biological and pharmacological conditions are uncommon, and cross-study comparisons are confounded by disease model, stage, route, dose, biodistribution, and duration of exposure. Its purpose is to identify mechanistic requirements that can be tested experimentally without inferring intrinsic superiority from heterogeneous studies.

### Scope and Evidence Base

We have prioritized recent peer-reviewed primary studies that directly tested glial transplantation, replacement, engineering, or EV use, while retaining older studies when they were foundational to mechanism or clinical development. Priority was given to studies that informed one or more variables central to the proposed framework, including the abundance and intrinsic competence of the relevant cell population, permissiveness of the lesion environment, anatomical distribution of the therapeutic target, and the required duration of therapeutic function. Translational reviews were used selectively for EV nomenclature, potency, manufacturing, pharmacology, and regulatory context [1,2,3,4,5,6,7,8]. Pharmacological recruitment of endogenous glia and depletion followed by endogenous repopulation are included as mechanistic comparators, not as direct evidence for donor-cell or EV therapy. Depletion/repopulation tests population renewal and possible state resetting, rather than reprogramming of a retained cellular population. Non-glial cell therapies are included only as mechanistic comparators that clarify the capabilities and limitations of glial cell and EV therapies. This is a focused narrative review, not a systematic evidence synthesis, as no claim of search completeness, formal risk-of-bias weighting, or quantitative efficacy comparison is made. The model/translation levels reported below describe the highest directly relevant experimental or clinical setting represented, not study quality or certainty of therapeutic benefit.

## 2. Replace–Reprogram–Relay: A Functional Framework for Platform Selection

Replace and Reprogram denote biological requirements, whereas Relay indicates a spatial/temporal delivery requirement. Replace addresses a missing or intrinsically defective cellular population or function. Reprogram refers to changing the functional state of surviving recipient cells without replacing the cellular population. It names the intended biological requirement, not an established molecular mechanism or a claim of durability. Relay specifies, when applicable, how therapeutic activity must be distributed and maintained or repeated and can accompany either biological mode. These requirements do not identify a therapeutic platform: cells may replace populations or modulate host cells, whereas EVs deliver molecular signals to recipient cells.

### 2.1. Distinct Therapeutic Capabilities of Living Glia and Extracellular Vesicles

Living glial cells and EVs differ most clearly in adaptive capacity and duration of action. A transplanted cell can sense ATP, cytokines, complement, debris, aggregated proteins, vascular injury, and metabolic changes. The cell can migrate, phagocytose, reprogram transcriptionally, and alter its secretory output as pathology evolves. Astrocytes and oligodendroglial cells also provide continuous homeostatic or structural functions that require a living cell. Unlike a living cell, an administered EV cannot respond after dosing because its membrane and cargo are fixed at harvest. That limitation can be useful as the product can be characterized before administration, delivered as a finite exposure, and redosed or stopped. Platform choice should therefore depend on whether benefit requires persistent adaptive cellular activity or a controlled molecular exposure, while recognizing that EV distribution and potency must be demonstrated for each product (Table 1).

### 2.2. From Biological Deficit to Therapeutic Strategy: Defining the Biological Mode and Exposure Requirement

**Scheme 1:**
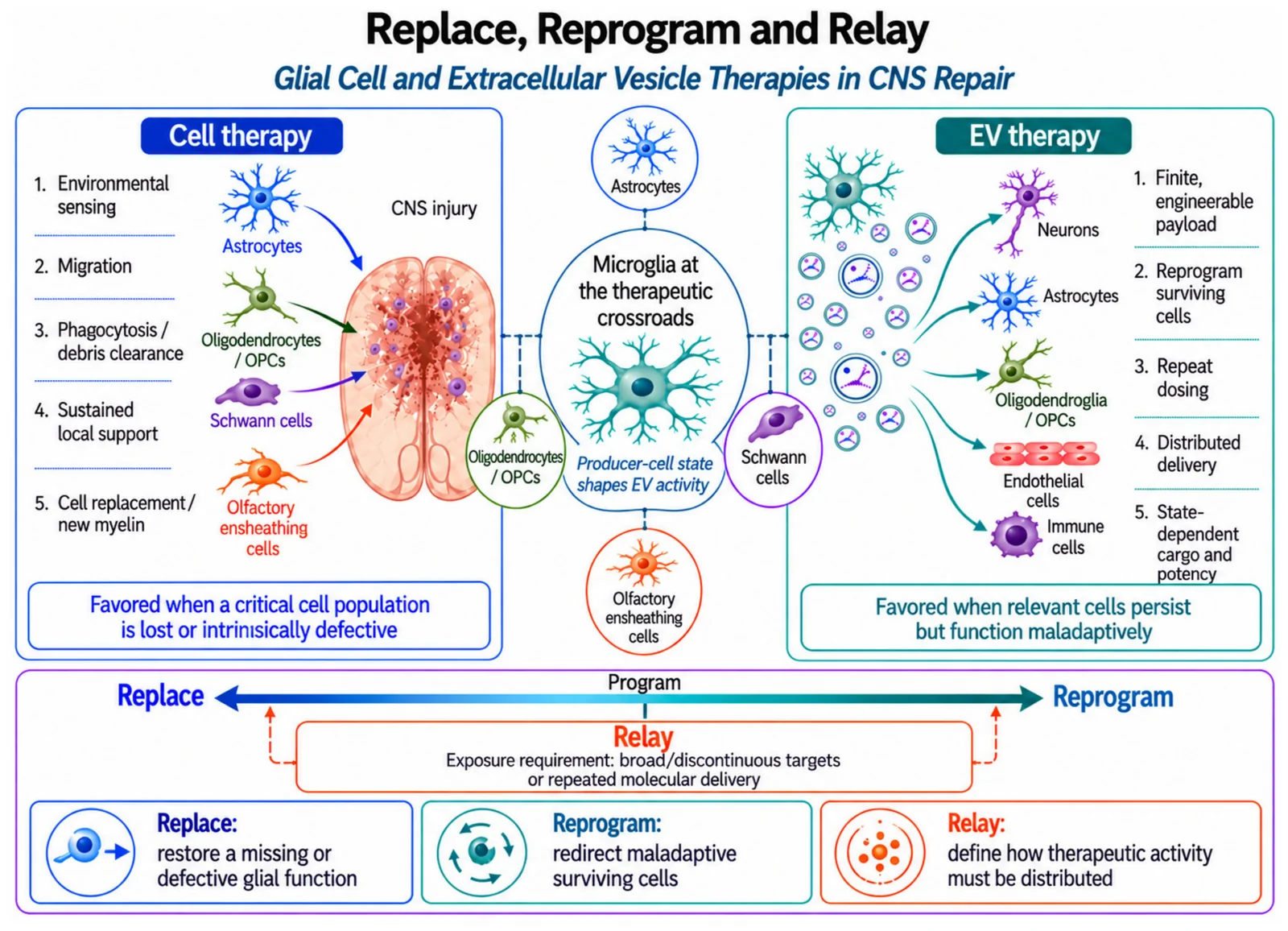
Microglia at the therapeutic crossroads between cell therapy and extracellular vesicle (EV) therapy. Cell therapy provides adaptive functions including environmental sensing, migration, phagocytosis, sustained support, and cellular replacement whereas EVs provide finite, engineerable molecular delivery. Microglial state shapes EV activity. Replace and Reprogram define biological requirements, whereas Relay defines an exposure requirement that can overlay either mode. Arrows indicate conceptual linkage, not sequence or efficacy hierarchy.

The Replace–Reprogram–Relay framework separates the biological deficit from the delivery problem (Scheme 1). “Replace” and “Reprogram” form the biological axis where loss or intrinsic dysfunction of a required cell population favors replacement, whereas preserved cells with reversible maladaptive function favor reprogramming. “Relay” is an orthogonal spatial/temporal delivery requirement, invoked when the therapeutic function must reach a broad or discontinuous anatomical territory or be delivered repeatedly.

These terms are linked and describe different therapeutic dimensions. A single intervention may satisfy more than one requirement: an engineered EV can deliver a payload to modulate recipient-cell function under a Reprogram requirement, and an engineered graft can replace or augment a deficient compartment while providing persistent local molecular output. Relay is not synonymous with EV therapy. Instead, it can be layered onto Replace or Reprogram, and pathology-responsive cells may themselves serve as relays after engraftment.

Evidence should be distinguished as modulation → phenotypic change → transcriptional reprogramming → durable state change. Here, modulation denotes altered signaling or activity; phenotypic change denotes a measured change in cellular behavior or function; transcriptional reprogramming requires a coordinated change in recipient-cell gene-expression programs; and durable state change requires persistence after the initiating exposure has ended. These are distinctions in evidence, not an obligatory biological sequence. Changes in proliferation, differentiation, apoptosis, inflammatory markers, or individual transcripts do not by themselves establish durable reprogramming.

**Figure 1:**
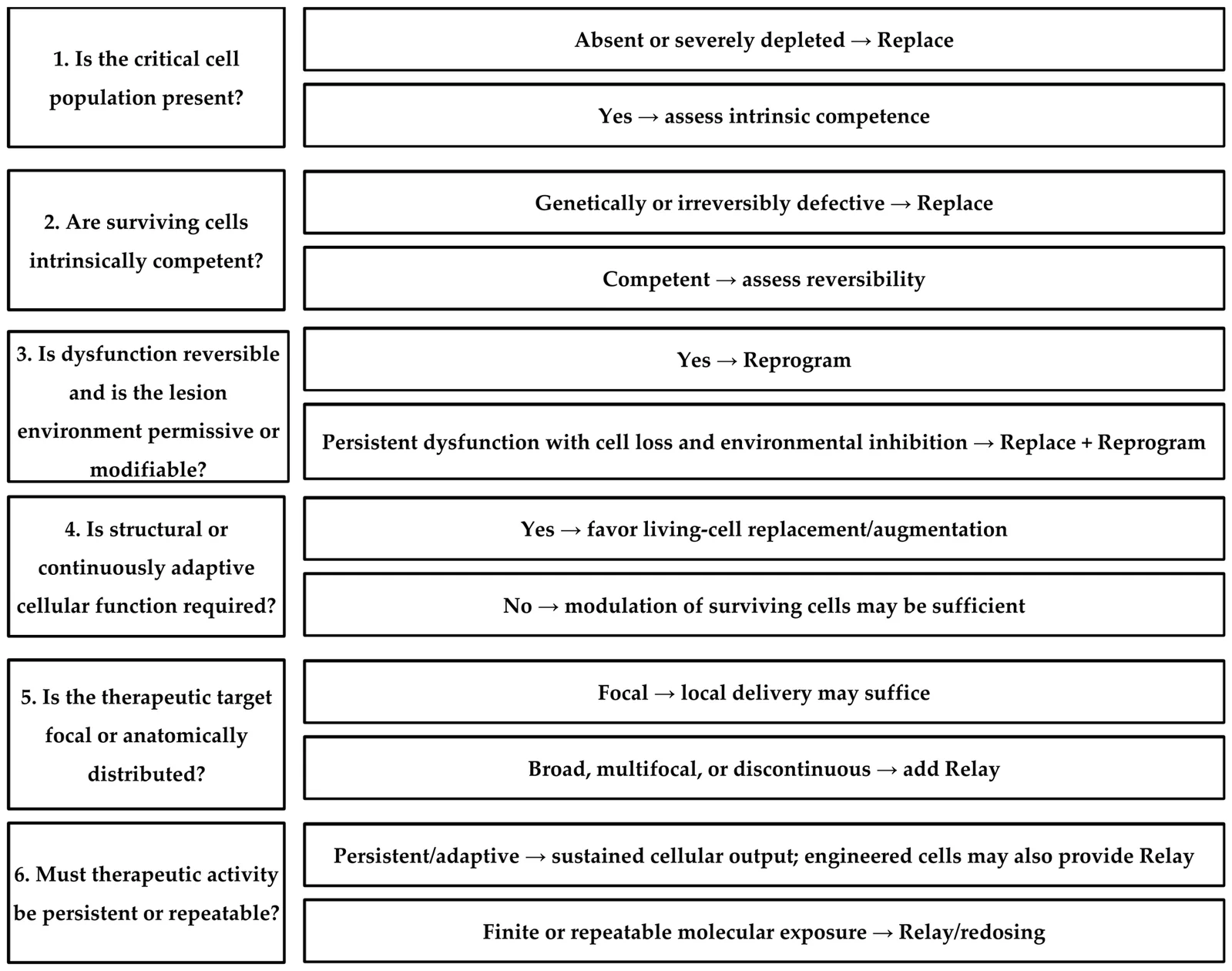
Prospective decision sequence for applying the Replace–Reprogram–Relay framework.

Five variables make the framework operational: (1) abundance of the relevant endogenous cells, (2) intrinsic competence of surviving cells, (3) permissiveness of the lesion environment, (4) anatomical distribution of the target, and (5) required duration of function. Severe cellular depletion or intrinsic/genetic dysfunction supports Replace, whereas preserved cells with modifiable dysfunction support Reprogram. An inhibitory environment may require additional modification, and broad or discontinuous targets or repeated molecular delivery add a Relay requirement. Concurrent cell loss and environmental inhibition may require combined Replace + Reprogram. These are mechanistic assignments, not rigid clinical thresholds. Prospectively, these variables can be applied as a sequential decision pathway to assign the dominant biological and exposure requirements (Figure 1).

Finite EV exposure and lasting therapeutic benefit are compatible, but neither implies durable recipient-cell reprogramming. A brief exposure may preserve vulnerable tissue or initiate repair whose consequences persist after EV clearance, without maintaining a new cellular state. Alternatively, benefit may depend on repeated dosing or sustained delivery. A claim of durable state change requires longitudinal molecular and functional measurements after dosing stops and residual EV-associated cargo activity is accounted for. Persistence of behavioral improvement alone is insufficient.

The intervention is assigned a dominant biological mode (Replace or Reprogram, or both when justified) and an exposure requirement (Relay, when applicable). Relay specifies the spatial and temporal delivery requirement rather than a separate biological mechanism. In Figure 1 and Scheme 1, Reprogram denotes this biological objective while the molecular mechanism and persistence of the recipient-cell response require separate evidence.

## 3. Microglial State Heterogeneity and Parent-Cell State as Determinants of Therapeutic Function

M1/M2 terminology remains common in older transplantation and EV studies because many experiments used cytokine-defined M1- or M2-like conditions [20]. It is not an adequate description of in vivo microglial biology. Single-cell, spatial, and epigenomic studies of microglial state diversity and nomenclature identify overlapping homeostatic, interferon-responsive, proliferative, lipid-associated, phagocytic, and disease-associated states that vary with region, age, sex, genotype, and pathology. Complementary work comparing EVs from mouse and human microglial cell lines highlights species- and model-associated differences in vesicle cargo [21,22,23,24,25,26,27,28,29,30]. In this review, M1/M2 terminology is retained only when reporting the original design of a cited experiment. Where conditioning is known, it is named directly; otherwise, a historical M2 label is explicitly attributed to the original study. “A1-like reactive astrocytes” is likewise retained only as a study-specific descriptor, not as a discrete or universally neurotoxic astrocyte class.

Chhatbar and colleagues integrated more than one million CNS myeloid cells from more than 30 physiological and pathological settings into 27 superclusters and 192 clusters, showing both cross-species conservation and strong context dependence [31]. Eggen and Kooistra likewise describe microglia as a spectrum of dynamic states rather than two fixed activation programs [32]. Therapeutically, an in vitro phenotype cannot be assumed to persist after transplantation into SCI, chronic active MS lesions, or amyloid-rich brain tissue.

The same state dependence applies to microglial EVs. IL-4 exposure, interferon signaling, oxidative or metabolic stress, senescence, and contact with misfolded proteins by the producer cell can change vesicle cargo and activity despite a common cellular origin. Producer-cell state forms part of the biological identity of an EV preparation. Particle concentration, size, and canonical EV markers remain necessary descriptors, but they do not capture state-dependent composition or potency [33,34,35,36].

## 4. Microglial Therapeutic Platforms: Transplantation, Niche Replacement, Engineering, and EV-Based Modulation

### 4.1. Transplanting Repair-Associated Microglia After CNS Injury

In mouse SCI, Kobashi and colleagues found that IL-4-conditioned microglia improved locomotor recovery relative to control or pro-inflammatory grafts and increased repair-associated Arg1, Mrc1, and Igf1 expression together with retrograde axonal transport [37]. Although the original study used an M1/M2 framework, the key result is that ex vivo conditioning can bias transplanted microglia toward a repair-supportive program.

Li and colleagues showed that neonatal microglia contribute to the nearly scar-free repair of the neonatal mouse spinal cord by transiently producing fibronectin and protease inhibitors that help organize the lesion and resolve inflammation. Transplantation of neonatal microglia into injured adult spinal cord reduced scar-associated matrix deposition and supported axonal growth. Adult microglia could acquire part of this reparative activity after pretreatment with E64 and Serpina3n before and at the time of transplantation [38]. The therapeutic value of a microglial graft depends on the repair program carried by the donor cells, where graft presence alone is insufficient.

Donor-cell engineering extends this strategy. Microglia overexpressing miR-145a-5p produced greater locomotor improvement than unmodified IL-4-conditioned cells after mouse SCI, with less inflammatory signaling, neuronal damage, and glial scar formation. miR-145a-5p suppressed TLR4/NF-κB signaling and helped maintain a repair-associated state [39]. The data remain preclinical but show that the therapeutic program of a microglial graft can be modified before delivery.

Local transplantation should be distinguished from replacement within the microglial niche. Local transplantation after injury can alter inflammation, matrix remodeling and axonal responses without substantially displacing the endogenous microglial population. Replacement requires donor-derived cells to gain access to the microglial niche, survive, proliferate and ultimately occupy a substantial fraction of the resident CNS myeloid compartment. Experimental studies have shown that depletion or conditioning of the endogenous microglial niche can greatly increase donor-cell engraftment. For example, Xu and colleagues developed strategies in which depletion of resident microglia combined with transplantation of microglia, bone marrow-derived cells or peripheral blood-derived cells produced substantial regional or CNS-wide replacement [10]. Notably, directly transplanted microglia retained a more microglia-like phenotype, whereas cells derived from bone marrow or peripheral blood remained relatively more macrophage-like, underscoring the importance of donor origin.

CNS entry does not make a peripheral macrophage equivalent to a resident microglial cell. After endogenous microglial depletion, circulating monocytes can occupy the niche and acquire microglial morphology and parts of the transcriptional and epigenetic program, yet engrafted cells retain features that distinguish them from embryonically derived microglia [40,41]. Donor ontogeny and recipient-niche signals therefore jointly shape the post-transplant phenotype. We distinguish replacement by donor microglia from repopulation of the microglial niche by donor-derived macrophages. Niche occupancy and restoration of selected functions do not, by themselves, establish microglial identity.

Recently, replacement platforms have sought to address conditioning more directly. Engineering human iPSC-derived microglia to express an inhibitor-resistant CSF1R variant allowed donor cells to expand selectively while endogenous microglia were depleted with a CSF1R inhibitor, eventually producing extensive replacement in experimental mouse brains [42]. More recently, brain-restricted conditioning combined with transplantation of committed myeloid progenitors achieved high-efficiency allogeneic repopulation of the microglial niche without systemic myeloablation in mice [43]. These approaches were developed primarily for genetic and neurodegenerative disorders rather than traumatic CNS injury, but they establish principles that are likely to be relevant to regenerative applications.

### 4.2. Creating a Replacement Niche: Conditioning and Donor Ontogeny

A central obstacle to replacement within the microglial niche is that the adult microglial compartment is normally closed to substantial colonization by circulating myeloid cells. Resident microglia are long-lived, self-renew locally, and occupy a tightly regulated tissue niche. Brain-penetrant CSF1R inhibitors such as PLX3397 and PLX5622 can eliminate most resident microglia experimentally [44,45], but depletion is reversible. After inhibitor withdrawal, residual endogenous microglia proliferate rapidly and can restore the compartment within days [45]. Depletion creates only a transient niche, requiring donor cells to engraft and expand before endogenous microglia repopulate.

Donor resistance to the conditioning agent can shift niche competition toward the graft. A G795A human CSF1R variant preserved receptor function while conferring inhibitor resistance, allowing iPSC-derived microglia to expand during PLX3397-mediated host depletion and achieve extensive brain-wide replacement [42]. Homologous murine Csf1r G793A strategies subsequently supported widespread engraftment after intraparenchymal or intracisternal delivery and CNS entry of resistant hematopoietic or myeloid cells after peripheral administration [46,47]. This converts a transient depletion window into a selective niche for donor cells.

Other strategies avoid conventional systemic myeloablation. Chen and colleagues developed a repeated depletion-transplantation approach, termed tricyclic microglial depletion for transplantation, in which cycles of PLX3397-mediated depletion created sufficient niche availability for transplanted primary microglia to achieve broad replacement. In mouse models of Sandhoff disease and TREM2 R47H-associated amyloid pathology, replacement with functionally competent microglia attenuated disease-associated changes [48]. Although described as a non-conditioning strategy because irradiation or chemotherapy was not required, repeated CSF1R-mediated depletion itself substantially alters the microglial compartment and should still be considered biologically when interpreting therapeutic effects.

Conditioning can also be restricted to the CNS. Mader and colleagues recently showed that committed Sca1-negative myeloid progenitors could efficiently repopulate the brain following intracerebral delivery and developed a brain-restricted conditioning strategy that avoided conventional systemic myeloablation and long-term peripheral donor-cell engraftment [43]. The approach produced high levels of allogeneic repopulation of the microglial niche and corrected neurological disease in a mouse model of Sandhoff disease. Human pluripotent stem cell-derived myeloid progenitors also showed substantial engraftment capacity under brain-restricted conditioning. These data suggest that extensive replacement may not require permanent hematopoietic chimerism or the toxicity associated with conventional hematopoietic stem cell transplantation.

The conditioning regimen, however, is not simply a technical prelude to transplantation. Irradiation and cytotoxic chemotherapy can alter the blood–brain–barrier, induce tissue injury and facilitate recruitment of peripheral myeloid cells. CSF1R inhibitors are considerably more selective, but they are also not biologically neutral: CSF1R signaling is shared by other myeloid populations, and CSF1R inhibition can affect border-associated macrophages and peripheral monocyte production under some conditions. Moreover, depletion followed by endogenous repopulation can itself modify disease phenotypes. Therapeutic effects observed after replacement need to be separated experimentally from those caused by depletion or conditioning alone, ideally by including conditioning-only and depletion-repopulation controls.

Donor ontogeny adds another layer of complexity. Cells of bone marrow or circulating monocyte origin can efficiently occupy a depleted microglial niche and acquire ramified morphology and expression of many microglial genes. Nevertheless, several studies have shown that these cells do not necessarily become molecularly equivalent to yolk-sac-derived microglia. Engrafted peripheral macrophages retain transcriptional, epigenetic and functional differences even after prolonged residence in the CNS [41,49,50]. Bennett and colleagues demonstrated that the CNS environment can induce components of the microglial program in macrophages from several sources, but that yolk-sac origin provides developmental information required for the most complete acquisition of microglial identity [49]. Similarly, bone marrow-derived macrophages can persist for long periods in the brain while maintaining distinct chromatin accessibility and responses to inflammatory challenge [41,50].

Recent work further resolves the contribution of donor ontogeny. Bastos and colleagues showed that adult monocytes can efficiently replace depleted brain macrophage populations and form long-lived monocyte-derived macrophages within the microglial niche, but these cells retain an identity distinguishable from embryonically derived microglia. In contrast, fetal liver monocytes possessed a distinct epigenetic state and, after engraftment, could develop a much more complete SALL1-positive microglial identity [51]. The findings suggest that developmental timing within the hematopoietic lineage can determine how completely a donor cell responds to instructive signals from the CNS niche.

Successful replacement should be judged by more than engraftment percentage. A high percentage of donor-derived IBA1-positive or TMEM119-positive cells is not, by itself, sufficient evidence that the resident microglial compartment has been functionally reconstructed. Replacement studies should establish the extent and anatomical distribution of donor engraftment, confirm acquisition of appropriate homeostatic transcriptional and epigenetic programs, including the transcriptional regulator SALL1 and identity-associated markers such as P2RY12 and TMEM119, and demonstrate maintenance of appropriate surveillance, phagocytic, and inflammatory responses within diseased or injured tissue. The therapeutic product is determined jointly by the donor cell, the niche created for it and the CNS environment into which it is introduced.

### 4.3. Genetic Microgliopathies: The Strongest Case for Replacement

CSF1R-associated microgliopathy, including adult-onset leukoencephalopathy with axonal spheroids and pigmented glia (ALSP), provides a compelling rationale for replacing the defective microglial compartment because the resident CNS myeloid population itself carries the disease-causing mutation. Suppressing downstream inflammation may modify the lesion environment, but it leaves a genetically abnormal, self-renewing population in place.

In a humanized CSF1R-related leukoencephalopathy model, transplanted human iPSC-derived microglial progenitors reduced white-matter pathology, axonal spheroids, astrogliosis, and calcification. CRISPR-corrected patient-derived microglia also improved established disease features [52]. Wu and colleagues subsequently reported eight patients with CSF1R-associated microgliopathy treated by bone-marrow transplantation, with disease progression reported to halt during 24 months of follow-up [14]. The uncontrolled cohort is small, and transplantation carries substantial clinical burden, so these data support feasibility and biological plausibility rather than definitive efficacy. The hematopoietic transplantation cohort should not be interpreted as evidence that donor-derived macrophages acquire an identity equivalent to resident microglia.

Krabbe disease provides a second monogenic example. In the twitcher (Galc^W355^*) mouse model, genetic microglial depletion followed by direct CNS delivery of healthy monocytes yielded donor-derived macrophages occupying more than 80% of the microglial compartment. Perinatal treatment normalized disease-associated macrophage transcriptional states, improved neuropathology, and approximately doubled average survival [53]. The study remains preclinical but extends the rationale for replacing a dysfunctional CNS myeloid compartment beyond CSF1R-associated disease. This is macrophage-mediated repopulation of the microglial niche and functional correction of the CNS myeloid compartment, not evidence that transplanted monocytes become ontogenetically equivalent to resident microglia.

### 4.4. Engineered Microglia as Pathology-Responsive Therapeutics

Microglial replacement becomes considerably more interesting when the donor cell is engineered to perform a function that is absent or inadequate in the host. Microglia continuously survey the CNS and undergo substantial transcriptional remodeling in response to local tissue damage, protein aggregates, demyelination, and other pathological cues. These responses are heterogeneous and cannot be reduced simply to a proinflammatory state. Instead, they provide an opportunity to couple therapeutic activity to disease-associated microglial programs, potentially concentrating treatment in regions where pathology is greatest while limiting constitutive exposure of the rest of the CNS.

Chadarevian and colleagues engineered human iPSC-derived microglia to express the Aβ-degrading enzyme neprilysin under a plaque-responsive CD9 regulatory program [54]. Localized and widespread engraftment reduced biochemical Aβ measures in an Alzheimer disease mouse model, but reductions in plaque burden, dystrophic neurites, and astrogliosis with neuronal preservation in the plaque-dense subiculum required broad CNS engraftment [54]. Efficacy therefore depended on both the engineered payload and access to regions with high pathological burden.

The study also showed that transplanted human microglia adopted different transcriptional responses when exposed to amyloid plaques, experimental demyelination, or breast cancer brain metastases [54]. Although therapeutic payloads were not separately developed for each of these conditions, the findings suggest that disease- or lesion-associated transcriptional programs could potentially be used to regulate different therapeutic outputs. In principle, engineered microglia might deliver aggregate-degrading enzymes in proteinopathies, trophic or repair-promoting factors in areas of tissue injury or demyelination, or locally acting immunomodulatory molecules in inflammatory lesions. The challenge will be identifying regulatory programs with sufficient pathological selectivity, because many microglial response genes are shared across injury, inflammation, aging, and neurodegeneration.

Engineering can also change what microglia recognize and remove, rather than only what they secrete. Heiss and colleagues expressed anti-amyloid chimeric antigen receptors (CARs) in human iPSC-derived microglia and found that selected constructs markedly enhanced the uptake of Aβ1-42. The most effective constructs produced approximately sixfold greater Aβ internalization than control cells, and the effect depended on intracellular CAR signaling and was also reproduced in primary mouse microglia [55]. These experiments were performed primarily in vitro and do not yet establish therapeutic efficacy after CNS transplantation. Nevertheless, they illustrate a conceptually distinct form of microglial engineering in which the recognition and phagocytic machinery of the cell is redirected toward a defined pathological substrate.

A related strategy is to restore a microglial function lost through a disease-associated genetic defect. In Trem2-deficient mice with amyloid pathology, systemic hematopoietic-cell transplantation followed by enhanced repopulation of the microglial niche by donor-derived myeloid cells introduced TREM2-competent myeloid-derived cells into the brain and restored important aspects of microglial function [56]. This was not a pathology-responsive synthetic circuit, but it showed that replacement with genetically competent cells can correct a functionally important microglial deficit.

Brain-restricted allogeneic repopulation of the microglial niche with committed myeloid progenitors also rescued disease in Hexb-deficient mice, a model of Sandhoff disease [43]. The donor population restored a missing microglial function while avoiding conventional systemic myeloablation and long-term peripheral hematopoietic engraftment. Together with TREM2-replacement studies, this defines a continuum from replacing cells within the defective microglial compartment to correcting specific functions or adding new therapeutic capabilities.

Regulation of the engrafted population will be a major translational requirement. Engineered microglia are attractive precisely because they can migrate, proliferate, respond to pathology, and alter their functional state in vivo, but these properties also make the final product more difficult to define after administration. Therapeutic output, spatial distribution, proliferation, genomic stability, and disease-induced state transitions will need to remain within acceptable limits over prolonged periods. Pathology-responsive promoters will require scrutiny because a regulatory program activated by the intended disease may also be engaged during infection, trauma, aging, or unrelated neuroinflammatory events. For constructs that produce potent secreted proteins or substantially alter phagocytic behavior, excessive or prolonged activity could itself become detrimental.

Inducible expression systems, genetically encoded elimination or safety switches, restricted proliferative capacity, and approaches that reduce immune recognition may become important components of future engineered microglial products. Most of these safeguards have not yet been extensively validated in therapeutic microglial replacement models and should be viewed as design priorities rather than established solutions. The next stage of the field will require demonstrating not only that engineered microglia can reach pathology and perform an intended function, but also that this activity remains appropriately regulated as the transplanted cells persist and adapt to a changing CNS environment.

### 4.5. Microglial EVs as State-Dependent Molecular Modulators

A microglial EV product captures selected components of microglial secretory biology without transplanting the responsive cell itself [57]. This approach is most relevant when neural, vascular or immune cells remain present but can be shifted toward a more favorable functional state. The therapeutic literature is still largely preclinical, however, and studies vary substantially in producer-cell phenotype, EV isolation, dose, route, and potency assessment.

## 5. Applying Replace–Reprogram–Relay Across CNS Injury and Demyelinating Disease

The following disease sections apply the framework rather than treating transplantation and EV delivery as interchangeable modalities.

### 5.1. Spinal Cord Injury

#### 5.1.1. Microglial EVs: Preserving and Modulating Surviving Tissue

SCI provides a useful example because the therapeutic target evolves with injury progression. Immediately after injury, during the acute phase, the therapeutic problem is largely one of preserving tissue that is damaged but not yet irreversibly lost. Hemorrhage, vascular disruption, excitotoxicity, oxidative stress, ionic imbalance, and innate immune activation place neurons, oligodendrocytes, axons, and vascular cells at risk during the first hours and days. At this stage, a therapy does not necessarily need to replace cells to have a meaningful effect. Limiting secondary injury, stabilizing the vasculature, reducing neuronal death, or changing the inflammatory response may preserve neural tissue that would otherwise be lost. For example, genetic deletion of transglutaminase 2 (TG2) or pharmacological treatment with cystamine improved tissue preservation and locomotor recovery in experimental SCI. The knockout experiments additionally demonstrated reduced fibronectin and chondroitin sulfate proteoglycan deposition, reactive gliosis, and markers associated with endoplasmic-reticulum, oxidative, and mitochondrial stress [58]. Similarly, early pharmacological modulation of endogenous lesion-associated myeloid cells with propentofylline and IL-4 improved locomotor recovery, reduced chronic p38 MAPK and NF-κB signaling and lesion cavitation, and increased ARG1- and CD206-associated myeloid responses [59]. Together with other approaches that limit secondary inflammatory injury, oxidative stress, or inhibitory extracellular-matrix remodeling [60,61,62], these studies illustrate how modifying the endogenous injury environment can preserve surviving tissue without requiring cellular replacement. As the lesion matures, however, the balance shifts. Myelin and axonal degeneration, persistent macrophage and microglial activation, astrocytic and fibrotic remodeling, extracellular matrix deposition, and cavitation leave progressively less intact tissue available for rescue. A treatment that is well suited to preserving vulnerable cells during the first several days may be poorly equipped to replace the myelinating cells, bridge a cavity, modify inflammation and the immune response, dampen scar formation, or reconstruct disrupted neural circuitry months or years later.

Much of the experimental literature on microglial EVs fits the first of these therapeutic objectives. Peng and colleagues administered microglia-derived EVs systemically during the early period after contusive SCI and found reduced oxidative stress within the injured cord, protection of spinal microvascular endothelial cells, increased vascular regeneration, and improved functional recovery. These effects were associated with activation of the Keap1/Nrf2/HO-1 antioxidant pathway [63]. The principal target in this case was not replacement of vascular or neural cells, but preservation of an endogenous vascular compartment exposed to the secondary injury environment. A related study showed that microglial exosomal miR-151-3p reduced neuronal apoptosis and promoted axonal growth by targeting p53 and modifying p53/p21/CDK1 signaling [64]. Again, the therapeutic substrate was largely tissue that remained present but was vulnerable to continuing injury.

The state of the EV-producing microglia also matters. EVs derived from anti-inflammatory microglia have shown greater reparative activity than those obtained from unconditioned microglia in several SCI models. For example, EVs enriched in miR-672-5p from microglia described as M2-like in the original study reduced neuronal pyroptosis by suppressing AIM2/ASC/caspase-1 signaling and were associated with greater axonal preservation and functional recovery [65]. More recently, Zhang and colleagues showed that intravenously delivered EVs from IL-4-conditioned microglia reduced the reported A1-like reactive astrocyte response, decreased lesion size, preserved neurons and axons, and improved motor function in mice. Suppression of NF-κB signaling and reduced nuclear translocation of p65 in astrocytes were implicated in this response [66].

Engineering has begun to address a second limitation of EV therapy, which is getting sufficient biologically active vesicles to the injured cord. Kong and colleagues modified microglia described as M2-like in the original study to produce Angiopep-2-bearing EVs and further conditioned the producer cells with melatonin. The resulting M-Ang2-EVs showed enhanced accumulation at the injured spinal cord, where LRP1 expression was increased after injury. Treatment was associated with increased microglial/macrophage clearance of sphingomyelin-containing myelin debris, greater axonal elongation and remyelination, and improved blood–spinal cord barrier integrity [67]. A subsequent strategy combined Angiopep-2-mediated barrier penetration with RGD-mediated targeting of lesion-associated neovasculature and curcumin conditioning of the EV-producing cells. These dual-targeted C-A/R-EVs shifted microglial transcriptional states toward reparative populations, enhanced myelin-debris phagocytosis, reduced inflammatory microglial populations, promoted axonal repair, and improved blood–spinal cord barrier integrity [68]. Such studies use engineered targeting to modify EV distribution without conferring active migration, but their principal action is still to alter the behavior of cells that are already present in or around the lesion.

Improvements in locomotor scores or axonal markers after early treatment should not automatically be interpreted as reconstruction of lost spinal tissue. In many studies, the dominant effect is more appropriately viewed as neuroprotection, vascular preservation, suppression of secondary injury, or enhancement of endogenous repair.

#### 5.1.2. Other Cellular Transplantation Approaches for SCI Repair

Transplantation of other cell types has shown promise for promoting repair after SCI, although outcomes vary substantially according to the cell population used, timing of transplantation, and biological state of the lesion environment. Human embryonic stem cell-derived oligodendrocyte progenitor cells (OPCs) transplanted 7 days after SCI differentiated into oligodendrocytes, enhanced remyelination, and improved locomotor recovery. In contrast, transplantation of the same cells 10 months after injury resulted in graft survival and differentiation but did not significantly increase remyelination or improve locomotor function [15]. Thus, even direct replacement of the myelin-forming lineage may be insufficient once the lesion has matured, underscoring the difficulty of extrapolating therapeutic efficacy observed in subacute SCI to established chronic injury.

Other studies indicate that cell transplantation can be more effective in chronic SCI when combined with interventions that modify the inhibitory lesion environment. Nori and colleagues combined human oligodendrogenic neural progenitor cells with sustained delivery of chondroitinase ABC using a biomaterial-based approach designed to reduce chondroitin sulfate proteoglycan-mediated inhibition within the chronic lesion. This combination enhanced graft survival and oligodendrocyte differentiation, supported remyelination of spared axons, increased synaptic connectivity, and improved behavioral recovery [69]. Similarly, in another chronic SCI model, neural progenitor cell transplantation alone was insufficient to produce significant functional recovery, whereas transplantation combined with intensive rehabilitation increased host corticospinal axon growth into the graft and improved forelimb function [70]. These findings suggest that successful cell transplantation in chronic SCI may require both provision of a reparative cellular substrate and modification of the host environment to support graft survival, integration, and functional circuit remodeling.

Schwann-cell transplantation provides a clinically relevant example of this reparative strategy. Schwann cells can provide trophic support, myelinate CNS axons, and create a cellular substrate that supports axonal growth. In a Phase I study involving eight individuals with chronic cervical or thoracic SCI, autologous human Schwann cells were transplanted directly into the chronic lesion using a cavity-filling approach together with rehabilitation [71]. The study demonstrated the feasibility of autologous Schwann-cell transplantation and an acceptable initial safety profile, although it was not designed to establish therapeutic efficacy. Unlike systemically administered extracellular vesicles, transplanted cells are intended to persist within the lesion, interact directly with host axons and glia, provide sustained trophic support, and potentially contribute to remyelination and structural repair.

Collectively, these studies demonstrate that cell transplantation can support multiple components of SCI repair, including neuroprotection, remyelination, axonal growth, trophic support, and neural circuit reconstruction. However, they also emphasize that the therapeutic effectiveness of transplanted cells is strongly constrained by the chronic lesion environment. The chronic-SCI combination studies described above support coupling cell transplantation with modification of extracellular inhibition or activity-dependent rehabilitation. Related work on Schwann cells, biomaterials, and adipose-derived stem cells provides broader context for modifying inflammatory and growth-inhibitory environments in SCI and peripheral nerve repair [72,73,74].

### 5.2. Ischemic Stroke

#### 5.2.1. Microglial EVs and Modulation of the Post-Ischemic Glial State

Ischemic stroke presents a therapeutic landscape in which recovery often depends on restoring the function of surviving peri-infarct cells rather than replacing cells lost within the infarct core. The infarct core undergoes extensive and often irreversible loss of neurons, glia, and vascular elements, but the surrounding peri-infarct tissue is biologically different. Neurons, oligodendrocyte-lineage cells, astrocytes, microglia, and endothelial cells can remain present but functionally impaired by inflammation, oxidative stress, disrupted vascular support, demyelination, and altered intercellular signaling. EV-based approaches are plausible in this setting because a defined EV preparation may influence several components of the neurovascular unit without requiring long-term engraftment, provided that product-specific biodistribution and target-cell uptake are demonstrated.

Song and colleagues showed that exosomes derived from IL-4-conditioned BV2 microglial cells reduced neuronal apoptosis after ischemia and decreased infarct volume and neurological deficits in mice. miR-124 was enriched in these exosomes and contributed to neuroprotection through suppression of USP14 [75]. Similarly, EVs carrying miR-135a-5p from microglia described as M2 in the original study reduced neuronal injury by suppressing TXNIP and downstream NLRP3 inflammasome activation [76]. These effects are best interpreted as preservation of vulnerable host tissue, because the EVs modify signaling within neurons that survived the initial ischemic insult rather than supplying replacement cells.

During the subsequent repair phase, when endogenous progenitors are present but do not always complete differentiation efficiently, Raffaele and colleagues examined GPR17-expressing OPCs after cerebral ischemia and found that microglia and macrophages initially supported the OPC response but later developed a dystrophic, senescence-like phenotype with loss of their pro-resolving activity. Intracerebral administration of EVs from regenerative microglia limited this immune-cell senescence and promoted maturation of GPR17-positive OPCs at the lesion border. Transmembrane TNF carried by the EVs contributed to the pro-differentiating effect on OPCs [77]. The treatment did not supply new oligodendrocytes, but instead, it restored signaling that allowed an endogenous progenitor population already present around the infarct to progress toward repair.

EVs from IL-4-conditioned microglia have been shown to promote OPC proliferation, survival, and differentiation after cerebral ischemia, increase oligodendrogenesis and white-matter repair, and reduce brain atrophy [78]. Much of the effect depended on transfer of EV-associated miR-23a-5p to OPCs, with Olig3 identified as a downstream target [78]. The result shows that an endogenous progenitor pool can be recruited without supplying exogenous OPCs.

Microglial EVs can also act on endogenous neural stem and progenitor cells. Small EVs from IL-4-conditioned microglia promoted neural stem-cell proliferation and neuronal differentiation after transient middle cerebral artery occlusion [79]. miR-25-3p and miR-93-5p were enriched in these EVs and contributed through regulation of TGFBR, PTEN, and FOXO3 signaling [79]. Microglial EVs may therefore support post-stroke repair by protecting differentiated neurons, promoting oligodendrocyte-lineage repair, and altering endogenous progenitor behavior.

Microglial vesicles can also act across the neurovascular unit. Zhang and colleagues described a migrasome-enriched EV fraction derived from IL-4-conditioned BV2 microglial cells that was taken up by microglia, astrocytes, neurons, and microvascular endothelial cells after experimental ischemia [80]. Treatment reduced neuronal apoptosis and abnormal astrocytic activation, promoted a more reparative microglial state and angiogenesis, improved cerebral blood flow, and reduced infarct volume and edema. These effects were associated with restoration of microglial mitochondrial homeostasis through cAMP/EPAC1/Rap1 signaling [80]. Although migrasome-enriched preparations should not be assumed to be equivalent to conventional small-EV or exosome preparations, the study illustrates a potential advantage of vesicle-based therapy after stroke: the ability to influence several interacting cellular compartments rather than replacing a single lineage.

Microglial EV activity is not intrinsically reparative. EVs generated from IFN-γ-treated microglia and administered beginning 7 days after transient middle cerebral artery occlusion reduced neural stem-cell survival and neuronal differentiation, inhibited post-stroke neurogenesis, increased brain atrophy, and impaired neurological recovery [81]. miR-199a-5p was enriched in these EVs, and knockdown of EV-associated miR-199a-5p restored neurogenesis, with SIRT1 identified as a downstream target [81]. Thus, different microglial states can produce EVs that act on the same reparative process, neurogenesis, in opposite directions.

#### 5.2.2. Microglial Depletion and Endogenous Repopulation as a Mechanistic Comparator

Microglial depletion followed by endogenous repopulation is a mechanistic comparator for modifying the post-ischemic glial environment. After depletion, residual microglia proliferate and renew the endogenous population. Any resulting differences in phenotype or function may reflect population renewal, altered population composition, and state resetting within a changed tissue environment. This is distinct from changing the functional state of a retained cellular population and therefore is not direct evidence for Reprogram as defined here. Nor does it establish conventional molecular reprogramming, durable state change, or efficacy of donor-cell transplantation or EV therapy. Depletion/repopulation controls remain important for separating the effects of conditioning and endogenous renewal from those of a transplanted product.

#### 5.2.3. Other Cell Transplantation Strategies to Improve Outcomes After Ischemic Stroke

Cell transplantation becomes more compelling when ischemia has eliminated the relevant glial population or endogenous repair is quantitatively insufficient. After experimental white-matter stroke, human iPSC-derived glial-enriched progenitors biased toward an astrocytic fate migrated through injured white matter and matured predominantly into pro-repair astrocytes [82]. Treatment increased endogenous OPC proliferation and remyelination, promoted axonal sprouting, and improved motor and cognitive recovery [82]. The graft acted as a persistent glial population within tissue where endogenous support had been lost, consistent with a role beyond transient soluble signaling.

OPC transplantation provides a direct comparison with EV-mediated stimulation of endogenous OPCs. OPCs transplanted 7 days after transient cerebral ischemia reduced subsequent brain atrophy, improved white-matter integrity and behavioral recovery, and increased endogenous OPC proliferation and migration [83]. The grafts also promoted neurite growth and synaptogenesis through mechanisms involving CXCL12/CXCR4-dependent oligodendrogenesis and Netrin-1/DCC signaling [83]. Transplanted OPCs can enter the oligodendrocyte lineage while simultaneously acting as local signaling centers that recruit endogenous repair.

Human iPSC-derived Olig2-induced OPCs were transplanted into rats after transient cerebral ischemia [84]. The grafts promoted remyelination, reduced neuronal loss, improved neurological recovery, and attenuated spatial-memory deficits [84]. These data support cell transplantation when oligodendrocyte loss or persistent white-matter damage exceeds endogenous repair capacity, while recognizing that grafted progenitors can also influence vascular, neuronal, and endogenous progenitor responses through paracrine signaling.

### 5.3. Multiple Sclerosis and Remyelination

Multiple sclerosis (MS) presents a different therapeutic problem from disorders in which a resident glial population is intrinsically defective and must be replaced. Human MS tissue studies show lesion-associated glial and neural abnormalities, including altered enzyme expression, such as TG2, and extracellular-matrix-associated changes, including fibronectin [85,86]. Complementary tissue-profiling, spatial, and single-cell studies characterize lesion-associated cellular and molecular changes. Spatial and single-cell analyses identify interacting lymphocyte, microglial, astrocytic, and oligodendroglial programs, including abnormalities in complement activation, antigen presentation, lipid and iron handling, mitochondrial function, and cellular metabolism [85,87,88,89]. Thus, an important therapeutic distinction in progressive MS is whether surviving glial populations remain capable of repair but are maintained in maladaptive states, or whether a critical cell population has become sufficiently depleted or dysfunctional that cellular replacement is required.

#### 5.3.1. Microglial State as a Determinant of Remyelination

Microglia are particularly relevant to this distinction because their effects on demyelination and remyelination are strongly dependent on cellular state, lesion compartment, and timing. Chronic active lesion rims contain inflammatory and metabolically altered microglial populations, including interferon-responsive, lipid-processing, iron-associated, and TREM2-positive states [90,91,92]. These states are not phenotypically or functionally equivalent. Experimental disruption of microglial cholesterol efflux promotes lipid-storing inflammatory phagocytes, whereas manipulation of sterol metabolism can attenuate inflammatory demyelination [90,91]. Similarly, microglial depletion can produce opposing effects on remyelination depending on experimental context. Depletion during early focal remyelination reduces OPC recruitment and proliferation and subsequently impairs oligodendrocyte differentiation and myelin repair, whereas depletion of reactive microglia during recovery from cuprizone demyelination can increase differentiating oligodendrocytes and restore mature oligodendrocyte density [93,94].

#### 5.3.2. Microglial EV-Mediated Modulation of Remyelination

The biological activity of microglial EVs is determined by the phenotype of the donor microglia and by the cellular environment in which the EVs act. Lombardi and colleagues demonstrated in focal lysolecithin demyelination that EVs released by inflammatory microglia inhibited remyelination, whereas EVs derived from microglia conditioned toward a regenerative phenotype enhanced OPC recruitment and myelin repair [33]. Importantly, the detrimental effects of inflammatory microglial EVs were mediated substantially through astrocytes, which acquired properties that inhibited oligodendrocyte differentiation [33]. Microglial EVs can influence remyelination through direct effects on the oligodendroglial lineage and indirect modulation of other components of the glial microenvironment.

The reparative activity of microglial EVs also reflects coordinated actions of multiple cargo classes rather than a single regulatory molecule. Microglial and macrophage EVs contain the endocannabinoids anandamide and 2-arachidonoylglycerol, and EV-associated endocannabinoid signaling can promote OPC differentiation through cannabinoid receptors [95]. However, inflammatory macrophage-derived EV preparations can still inhibit OPC maturation despite containing potentially pro-differentiating lipids, emphasizing that the net biological effect of an EV preparation reflects the integrated activity of its cargo [95]. More recently, Raffaele and colleagues showed that EVs generated from IL-4-conditioned or mesenchymal-stromal-cell-conditioned regenerative microglia induced extensive transcriptional changes in differentiating OPCs, with substantial convergence on mitochondrial function and bioenergetic metabolism [34]. In contrast, EVs from inflammatory microglia produced a substantially weaker pro-differentiation response [34]. These data support a model in which appropriately conditioned microglial EVs promote remyelination by coordinating metabolic and differentiation programs in endogenous OPCs while simultaneously modifying the surrounding glial environment. The observed transcriptional responses support modulation of recipient-cell programs, but their persistence after withdrawal of EV exposure must be established before they are interpreted as durable reprogramming.

#### 5.3.3. Whole-Cell Replacement Within the Microglial Niche

Whole-cell replacement within the microglial niche represents a fundamentally different therapeutic strategy from EV-mediated modulation. Rather than attempting to modify the behavior of resident microglia indirectly, this approach seeks to reconstruct the lesion-associated myeloid compartment by introducing exogenous microglia or microglia-like cells with a more favorable functional state. In principle, such a strategy could be advantageous when endogenous microglia are persistently maladaptive, intrinsically dysfunctional, or insufficiently responsive to functional modulation.

A recent review proposed depletion of resident microglia followed by transplantation of iPSC-derived microglia as a potential strategy for progressive MS [96]. Direct therapeutic transplantation of human iPSC-derived microglia, however, has not yet been established in MS. The rationale has largely been extrapolated from microglial depletion/repopulation studies and from transplantation paradigms developed in other neurological disorders.

More recently, preclinical work has begun to provide proof-of-concept for cellular replacement of the myeloid compartment in experimental MS. Separately, Benito-Cuesta and colleagues demonstrated that allogeneic MHC-mismatched microglia-like cell replacement can be implemented in an MS-relevant experimental setting [97]. These findings provide initial proof-of-concept for replacement within the microglial niche by microglia-like cells in experimental MS, although the efficacy and translational feasibility of human iPSC-derived microglia in progressive MS remain unknown.

An additional complication is that microglial depletion itself can have context-dependent effects on remyelination. Depending on lesion stage and experimental model, depletion may either impair or facilitate oligodendrocyte regeneration [93,94]. Thus, removal of endogenous microglia cannot be assumed to produce a more permissive reparative environment. Any replacement strategy will need to separate the effects of conditioning or depletion from those of the transplanted cells themselves and determine whether the donor population acquires an appropriate disease-relevant phenotype after engraftment.

In progressive MS, where microglia are generally retained but occupy heterogeneous inflammatory and metabolically altered states, EV-mediated modulation has direct preclinical support in demyelination models for promoting oligodendroglial repair [33,34,95]. Whole-cell replacement may become more compelling when resident microglia prove persistently refractory to functional modulation or when irreversible cellular dysfunction is demonstrated. At present, however, microglial replacement should be regarded as an emerging preclinical strategy rather than an established therapeutic approach for MS.

#### 5.3.4. Other Cell-Mediated Repair Strategies to Promote Remyelination in MS

The same reprogram-versus-replace principle applies to the oligodendrocyte lineage. OPCs can persist within chronically demyelinated MS lesions even when their progression into mature, myelinating oligodendrocytes is markedly impaired. Kuhlmann and colleagues identified Olig2-positive progenitors in chronic MS lesions but relatively few cells undergoing active maturation, supporting a differentiation block as an important mechanism of remyelination failure [98]. In such lesions, supplying additional OPCs may not by itself correct the underlying problem, and the first therapeutic requirement may instead be to restore the ability of resident OPCs to differentiate and function within an inhibitory lesion environment.

Clinical studies provide evidence that endogenous oligodendroglial populations can remain therapeutically responsive, while also illustrating the importance of disease stage and lesion context. In the ReBUILD trial, clemastine, identified in preclinical studies for its ability to promote OPC differentiation, reduced visual-evoked-potential latency by approximately 1.7 ms per eye in individuals with relapsing MS and chronic demyelinating optic neuropathy [99,100]. Although modest, this physiological effect provided evidence consistent with remyelination in a chronically demyelinated human pathway without transplantation of new oligodendrocyte-lineage cells. This result supports the possibility of pharmacologically enhancing endogenous repair, but does not directly demonstrate OPC recruitment or histological remyelination in the participants and does not establish efficacy for either an EV- or cell-transplantation platform.

Importantly, the response to a remyelinating intervention cannot be assumed to remain constant across stages of MS. In the TRAP-MS platform study involving individuals with progressive disease, the clemastine arm was stopped after three of nine participants with follow-up met prespecified safety stopping criteria, and treated participants demonstrated increased disability-progression slopes accompanied by CSF proteomic evidence of increased purinergic signaling and pyroptosis-associated pathways [101]. The small sample precludes broad conclusions regarding remyelination therapy in progressive MS. The associated mechanistic experiments implicate clemastine-mediated potentiation of ATP/P2RX7 signaling and pyroptotic injury, rather than establishing that stimulation of OPC differentiation caused the adverse outcomes. Thus, the biological state of the recipient lesion must be considered alongside the regenerative potential of the targeted cell population.

A stronger rationale for transplantation emerges when the endogenous oligodendroglia compartment is substantially depleted, persistently refractory to differentiation, or quantitatively incapable of generating sufficient new oligodendrocytes to restore myelin. Experimental studies demonstrate that exogenous OPCs can survive and respond to demyelinated tissue. Thiruvalluvan and colleagues transplanted human iPSC-derived OPCs into a marmoset model containing MS-like inflammatory demyelinating lesions. The transplanted cells survived, migrated preferentially toward demyelinated lesions in the corpus callosum, and differentiated into oligodendrocytes capable of myelinating denuded axons [102]. This study provides important evidence for the biological feasibility of OPC replacement in an inflammatory demyelinating environment, although it does not establish that transplanted OPCs would engraft broadly or maintain reparative function within chronic progressive MS lesions in humans.

Additional transplantation studies reinforce the importance of both donor-cell state and recipient environment. In a chronic cuprizone model, transplanted OPCs increased myelin-associated measures and reduced CSPG4 immunoreactivity, but the magnitude of repair varied according to the environment from which the donor OPCs had been obtained [103]. Because cuprizone-induced demyelination lacks the adaptive autoimmune component of MS, these findings cannot be extrapolated directly to progressive MS. Nevertheless, they show that transplantation outcome is influenced by donor-cell identity, prior biological state, and the recipient microenvironment.

Neural stem-cell transplantation further illustrates that cell replacement and modulation of surviving host cells are not mutually exclusive mechanisms. Transplantation of iPSC-derived neural stem cells in experimental autoimmune encephalomyelitis was associated with reduced T-cell infiltration, decreased white-matter injury, and improved neurological function [104]. These effects cannot be attributed solely to replacement of cells lost through demyelination. Transplanted neural progenitors can also provide trophic and immunomodulatory signals that alter inflammatory responses and support endogenous neural and glial populations. A transplanted cell may function as both a structural cellular substrate and a persistent local source of paracrine signals.

Microglial EVs are particularly suited to lesions in which resident microglia, astrocytes, and OPCs remain present but occupy maladaptive inflammatory, metabolic, or differentiation states [33,34,95]. Whole-cell microglial replacement would represent a more radical reconstruction of the lesion myeloid compartment. Although recent preclinical work has begun to test microglia-like cell replacement in experimental MS, therapeutic transplantation of human iPSC-derived microglia remains largely unexplored in MS [96,97]. By contrast, transplantation of OPCs or neural progenitors becomes increasingly rational when endogenous oligodendroglial repair capacity is depleted or persistently inadequate [102]. In progressive MS, therapeutic choice should be anchored to the underlying biological deficit: whether surviving cells can be functionally modulated without population replacement or irreversible dysfunction of a critical cellular population necessitates replacement.

## 6. Pathogenic Microglial EVs as a Constraint on Therapeutic Design in Neurodegeneration

Pathogenic microglial EV biology does not imply that a manufactured microglial EV product will necessarily reproduce disease-associated signaling. Inflammatory exposure, protein aggregation, metabolic stress, aging, and disease-associated transcriptional programs can alter EV release as well as their protein, lipid, RNA, and surface composition. Isolation and enrichment methods can then further determine which of these heterogeneous populations are recovered.

### 6.1. Alzheimer’s Disease

Alzheimer’s disease illustrates the bidirectional nature of microglial EV biology. TREM2 on microglial EV membranes can influence vesicle secretion and Aβ binding, and these EVs enhance microglial Aβ uptake [105]. Thus, EV release can participate in an endogenous Aβ-clearance pathway as well as in pathogenic protein transfer, depending on microglial state and vesicle composition.

The same system can facilitate pathological spread. In an experimental tauopathy model, microglial depletion markedly reduced tau propagation, and inhibition of neutral sphingomyelinase 2, which reduced EV secretion in that system, also suppressed tau transfer in vitro and in vivo [106]. In an APP knock-in model, plaque-associated microglia released increased numbers of phosphorylated-tau-containing EVs, and microglial depletion reduced tau propagation [107] but simultaneously increased plaque burden and plaque-associated dystrophic neurites. Microglia can therefore contribute to Aβ containment while also providing a route for tau dissemination. Thus, blocking a pathogenic EV pathway is not equivalent to eliminating the producer cell.

Gabrielli and colleagues showed that large microglial EVs carrying Aβ altered dendritic spine morphology and impaired long-term potentiation. Following injection into the entorhinal cortex, the synaptic deficit subsequently appeared in the connected dentate gyrus. Notably, soluble Aβ42 alone and inflammatory EVs that lacked Aβ did not reproduce this propagation of synaptic dysfunction [108]. The result argues that pathogenicity was determined by the combination of EV carrier and cargo rather than by inflammation or Aβ exposure alone.

EV subtype adds another layer of complexity. Large microglia-derived EVs, termed macrosomes, inhibited Aβ aggregation, reduced plaque burden, and improved cognitive performance in an AD mouse model [109], whereas small EVs from the same broad cellular source slightly promoted Aβ aggregation and did not improve pathology. Proteomic comparison identified protective proteins enriched in macrosomes, including SMIM10L2B, which contributed to inhibition of Aβ aggregation [109]. Even EV populations released by the same cell type can exert opposing effects on the same pathological substrate.

### 6.2. Parkinson’s Disease and Synucleinopathy

Parkinson’s disease (PD) presents a related problem in which microglial uptake of pathological protein can be followed by its redistribution through EV-mediated signaling. Guo and colleagues showed that microglia exposed to α-synuclein preformed fibrils released EVs containing α-synuclein that induced aggregation in recipient neurons [110]. Injection of these EVs into mouse striatum produced propagation of phosphorylated α-synuclein along anatomically connected pathways and was accompanied by nigrostriatal neurodegeneration. The study also provided human evidence that CD11b-positive EVs isolated from the cerebrospinal fluid of patients with PD contained oligomeric α-synuclein and promoted α-synuclein aggregation in recipient neurons [110].

TLR2 appears to participate in this cycle. Xia and colleagues found that EVs from the plasma of patients with PD preferentially interacted with microglia and that vesicle-associated α-synuclein engaged microglial TLR2 [111]. TLR2-dependent uptake promoted microglial activation and provided a route through which internalized α-synuclein could subsequently be transmitted to neurons. Silencing TLR2 reduced α-synuclein pathology in vivo, supporting a model in which recognition, inflammatory activation, and subsequent vesicular transfer are mechanistically coupled rather than independent events [111].

Pathogenic activity is not restricted to the transfer of α-synuclein itself. Jiang and colleagues demonstrated that extracellular α-synuclein oligomers activate P2X7 receptor (P2X7R)-mediated PI3K/AKT signaling in microglia, promoting the release of EVs containing cathepsin L (CTSL). These EVs contributed to neuronal injury and death, whereas pharmacological inhibition of EV release, P2X7R blockade or knockdown, and CTSL inhibition significantly attenuated the associated neurotoxicity [112]. This shows that a disease-associated microglial EV can amplify neuronal injury through altered host–cell cargo even when the cargo is not the initiating aggregated protein.

Lewy body disease studies extend this mechanism to transfer of functional membrane proteins. Activated microglial EVs were found to be enriched in LAG3 and cholesterol and to transfer LAG3 to cholinergic neuronal membranes through an endosomal recycling pathway. This increased neuronal internalization of extracellular α-synuclein and aggravated α-synuclein deposition and cognitive abnormalities in an experimental model targeting the nucleus basalis of Meynert [113]. In this case, the EV did more than transport a toxic cargo: it altered the receptor composition of the recipient neuron and thereby changed its susceptibility to subsequent pathological protein uptake.

## 7. Extending Mechanistic Relevance Beyond Microglia

### 7.1. Astrocytes: Continuous Cellular Support Versus Vesicle-Mediated Modulation

Astrocytes exhibit a different limitation of a simple cell-versus-EV comparison because many of their essential functions are continuous and spatially organized. Astrocytes clear extracellular glutamate, regulate potassium and water homeostasis, supply metabolic substrates, maintain antioxidant defenses, participate in extracellular matrix remodeling, and interact with synapses and the neurovascular unit. Many of these functions depend on a living cell occupying the appropriate anatomical territory and maintaining membrane transporters, channels, metabolic pathways, and vascular endfeet. An EV can alter these processes in a recipient cell, but it cannot itself clear extracellular glutamate, buffer potassium, or provide continuous metabolic coupling to an axon.

SCI transplantation studies show why astrocyte-product identity matters. Astrocytes generated from glial-restricted precursors supported axonal growth and locomotor recovery more effectively than undifferentiated precursors [114]. In subsequent work, astrocytes differentiated from human glial progenitors with bone morphogenetic protein promoted neuronal survival, axonal growth, and recovery of volitional foot placement, whereas CNTF-differentiated astrocytes from the same precursor population did not produce comparable benefits [115]. Two populations classified broadly as astrocytes can produce markedly different transplantation outcomes.

Outcome also depends on injury model and host environment. In a contusion SCI model, human glial-restricted progenitors and their astrocyte derivatives survived, migrated extensively, and reduced cyst and scar formation, yet neither population produced a significant improvement in locomotor recovery [116]. These differences across studies emphasize that developmental stage, regional identity, differentiation protocol, maturation state, injury type, timing of transplantation, and the local lesion environment all influence graft behavior. The term “astrocyte” alone is insufficient to predict therapeutic function.

Astrocyte EVs are similarly state-dependent. In a recent quantitative proteomic study of SCI, EVs released by relatively homeostatic astrocytes reduced microglial activation and neuronal apoptosis while promoting axonal growth and oligodendrocyte precursor cell maturation. EVs generated by reactive astrocytes instead exacerbated inflammation and impaired functional recovery, with CCL7 identified as an important mediator of their adverse activity [117]. Because the producer-cell lineage was unchanged, what changed was the state of that cell and, consequently, the composition and activity of its EVs.

### 7.2. Astrocytes in ALS: Sustained Local Support Versus Broad Tissue Distribution

Building on Section 7.1, ALS is used here as a mechanistic example within the astrocyte-focused comparison, illustrating how the anatomical distribution of pathology and required duration of support inform cell- and EV-based therapeutic strategies. ALS provides strong evidence for non-cell-autonomous astroglial toxicity. Astrocytes from familial and sporadic ALS tissue reduce motor-neuron survival in vitro [118], and similar toxicity occurs in directly converted astrocytes from SOD1- and C9ORF72-associated as well as sporadic ALS [119]. Human neural progenitors derived from sporadic ALS iPSCs also differentiated into astrocytes after cervical spinal-cord transplantation and produced neuronal degeneration and motor abnormalities in mice [120]. These observations support strategies that suppress harmful astroglial states or restore healthier support functions.

Engineered GDNF-secreting human neural progenitor cells provide a clinical example. In a Phase 1/2a study, CNS10-NPC-GDNF cells were transplanted unilaterally into the lumbar spinal cord of 18 participants with ALS [121]. The primary safety endpoint was met, and postmortem analysis showed graft survival and continued GDNF production. In some participants, graft-derived support cells and GDNF expression persisted for as long as 42 months. The study was not designed to establish clinical efficacy, and benign neuromas near delivery sites were noted at autopsy, but it demonstrated prolonged local production of a biologically active trophic factor in the human spinal cord [121].

Persistence is both an advantage and a limitation. ALS affects motor systems distributed across the spinal cord, brainstem, motor cortex, and peripheral motor unit. A locally engrafted cell can provide sustained exposure within its anatomical territory, but it does not readily address disease across the entire neuroaxis. EVs offer a different exposure profile. Repeated dosing and distribution beyond a single injection site may be achievable, and EVs can carry proteins, RNAs, or therapeutic oligonucleotides into multiple recipient cell populations. The trade-off is that EVs do not provide the same continuous local output as a stable graft and may require repeated administration to maintain effective exposure.

ALS also shows why EV cargo cannot be inferred from the apparent phenotype of the producer cell. In C9ORF72 ALS, patient-derived induced astrocytes showed altered EV formation and miRNA cargo [122], including reduced miR-494-3p, a negative regulator of SEMA3A and other axonal-maintenance pathways. Their EVs impaired neurite networks and motor-neuron survival, whereas restoring miR-494-3p reduced SEMA3A and improved motor-neuron survival [122]. Pathogenicity therefore arose in part from loss of a normally protective regulatory cargo rather than enrichment of a toxic miRNA alone.

A different mechanism operates in SOD1-associated disease. EVs released by SOD1^G93A^ astrocytes reduced motor-neuron survival and neurite length and showed altered miRNA composition, including miR-155-5p. Inhibition of miR-155-5p prevented the adverse effects of these EVs on motor neurons [123]. The C9ORF72 and SOD1 studies show that astrocyte EV toxicity can arise through distinct cargo changes in different genetic contexts. They also caution against assuming that an EV fraction will reproduce the beneficial properties of an intact astrocyte or, conversely, that an astrocyte judged acceptable by conventional phenotypic markers will necessarily produce a therapeutically acceptable EV population.

### 7.3. Oligodendroglial Repair: A Structural Function Sets the Boundary

Myelin formation defines a clear boundary between cellular replacement and EV-mediated support. EVs can influence OPC survival and differentiation, modify inflammatory or metabolic signaling, and protect stressed axons. They cannot, however, extend oligodendroglial membrane around an axon and generate compact myelin. When the principal limitation is an insufficient number of competent myelin-forming cells, transplantation of an oligodendroglial lineage cell has a structural capability that no vesicle dose can reproduce.

Human embryonic stem cell-derived OPCs transplanted after cervical contusion injury survived, reduced lesion pathology and tissue loss, preserved motor neurons, and improved forelimb function [124]. These benefits should not be attributed exclusively to remyelination, because transplanted OPCs can also provide trophic support and alter the lesion environment. Nevertheless, the ability of the graft to generate oligodendrocytes and potentially restore myelin distinguishes it fundamentally from an acellular therapy.

The platform entered clinical testing as LCTOPC1, previously designated GRNOPC1 or AST-OPC1. In an open-label Phase 1/2a dose-escalation study, 25 participants with subacute C4-C7 AIS A or B SCI received a single intraparenchymal administration of 2 million, 10 million, or 20 million cells 21 to 42 days after injury [125]. The primary objective was safety. Two grade 3 serious adverse events were considered related to the injection procedure (cerebrospinal fluid leak) and tacrolimus immunosuppression (bacterial infection), respectively. No enlarging graft-associated masses or treatment-related spinal cord damage were identified during the reported follow-up, and neurological improvement was observed in a subset of participants. Because the study was uncontrolled and designed primarily to assess safety, these neurological changes cannot establish efficacy [125].

Long-term follow-up of the earlier thoracic SCI cohort provides an additional safety benchmark. Five participants with neurologically complete T3-T11 injuries received 2 million LCTOPC1 cells 7 to 14 days after injury. Follow-up extending to 10 years found no unanticipated serious adverse events attributed to the cell product, no progressive neurological decline, and no evidence of enlarging masses, additional cord damage, or syrinx formation [126]. These data are reassuring for long-term graft safety in a small cohort, although they do not resolve questions of efficacy or establish safety for larger doses and different injury settings.

CRISPR deletion of NRP1 made human embryonic stem cell-derived OPCs resistant to the chemorepulsive effect of SEMA3A found in chronic MS lesions [127]. Edited cells migrated more effectively and produced greater remyelination than controls in rodent focal-demyelination models incorporating SEMA3A to mimic an inhibitory feature of chronic MS lesions. Although not an SCI study, it shows that a structural cell therapy can be engineered both to supply a missing lineage and to overcome a defined inhibitory feature of the recipient lesion.

Oligodendrocytes also use EVs as part of normal axonal support. Neuronal glutamatergic activity can stimulate oligodendrocyte EV release, followed by neuronal uptake. Oligodendroglial EVs improve neuronal survival during cellular stress, establishing an activity-dependent glia-to-neuron communication pathway [128]. Later work has shown improved neuronal metabolic state and axonal transport during nutrient deprivation, whereas EVs from oligodendrocytes carrying genetic defects associated with secondary axonal degeneration were less effective [129].

### 7.4. Schwann Cells and Olfactory Ensheathing Cells as Complementary Repair Models

Schwann cells and olfactory ensheathing cells (OECs), although not CNS-resident or CNS-exclusive glia, respectively, in the same sense as microglia, astrocytes, and oligodendrocytes, provide complementary models for cell-versus-EV comparison. Both cell types normally operate in environments that support axonal growth, and both can modify an injured CNS when transplanted. Their transplantation literature also highlights an important distinction: some regenerative effects arise from trophic and immunomodulatory signals that can potentially be transferred by extracellular vesicles, whereas others depend on the continued physical presence, migration, and organization of the donor cells.

After peripheral nerve injury, Schwann cells adopt a repair program that supports neuronal survival, clears myelin debris, produces trophic and matrix signals, and forms longitudinal pathways for regenerating axons. In the injured spinal cord, transplanted Schwann cells can provide a growth-permissive substrate, support axonal ingrowth and plasticity, and myelinate suitable axons. These persistent cell–cell and cell–matrix functions cannot be reproduced fully by a transient EV preparation.

Autologous Schwann-cell transplantation has progressed into early clinical testing in SCI. In a Phase 1 dose-escalation study, six participants with neurologically complete subacute thoracic SCI received highly purified autologous Schwann cells generated from a sural nerve biopsy and transplanted into the lesion epicenter approximately 4–7 weeks after injury [130]. No cell therapy-related serious adverse events, additional spinal cord damage, mass lesions, or syrinx formation were identified during the initial one-year follow-up, establishing the feasibility of producing and delivering an autologous Schwann-cell product in the subacute period [130]. A subsequent Phase I study extended this approach to chronic cervical and thoracic SCI. Eight participants received autologous Schwann cells using a lesion cavity-filling strategy together with standardized rehabilitation. No serious adverse events attributable to the cell transplantation were reported [71]. Some participants showed neurological or imaging changes, but the small, uncontrolled safety study was not designed to establish efficacy.

These grafts act through more than trophic factor secretion. A viable Schwann-cell graft can occupy lesion space, interact continuously with regenerating axons and host glia, remodel extracellular matrix, and provide myelin when appropriate axonal substrates are present. These are inherently persistent cellular functions. At the same time, Schwann cells also release soluble factors and EVs that can reproduce selected components of their repair program without requiring long-term graft survival [131].

Lopez-Verrilli and colleagues showed that Schwann cell-derived EVs were selectively internalized by axons, shifted growth cones toward a regenerative morphology, reduced RhoA activity, and enhanced axonal regeneration in culture and after sciatic nerve injury [132]. EVs from skin precursor-derived Schwann cells likewise promoted motoneuron axonal growth and recovery from axotomy or oxygen-glucose deprivation through Akt/mTOR/p70S6K signaling [133]. In sensory neurons, small EVs from the same source promoted neurite growth and survival with evidence implicating the miR-21-5p/PTEN/PI3K/Akt signaling pathway [134]. These studies show that part of the Schwann cell-axon repair program can be transferred through vesicles without direct cell contact.

Schwann-cell EVs also modify non-neuronal components of injured spinal cord. Systemic Schwann-cell EVs increased astrocytic TLR2 signaling and reduced chondroitin sulfate proteoglycan deposition, whereas astrocyte-specific TLR2 deletion abolished much of the effect [135]. EVs containing MFG-E8 also altered macrophage/microglial responses through SOCS3/STAT3 signaling, reduced neuronal apoptosis, and improved functional outcomes, whereas MFG-E8 knockdown in producer cells diminished these effects [136]. These experiments provide direct cargo-linked evidence that Schwann-cell EVs can coordinate neuronal and glial repair responses.

OECs provide a related comparison. In the intact olfactory system, OECs accompany continuously regenerating olfactory axons as they extend from the peripheral olfactory mucosa into the CNS. This unusual location at a natural PNS-CNS interface contributed to their early development as a transplantation strategy for SCI. Experimental studies suggest that OECs can support axonal growth, modify inflammatory and astroglial responses, promote tissue preservation, and interact with endogenous Schwann cells within injured spinal cord tissue, although the relative contribution of these mechanisms varies substantially between preparations and injury models [137]. Autologous OEC-containing preparations have also undergone limited clinical investigation in chronic SCI. A small Phase 1 study of autologous olfactory mucosal OECs and olfactory nerve fibroblasts established feasibility and short-term safety, although the small cohort and combined rehabilitation paradigm precluded firm conclusions regarding efficacy [138].

Part of the OEC repair program can also be transferred through EVs. Human OEC-derived EVs contain a complex miRNA repertoire and have been shown in vitro to protect neural progenitor cells against oxidative injury, support neurite extension and cell migration, and enhance endothelial tube formation [139]. More directly in SCI, Fan and colleagues showed that OEC-derived EVs were taken up by microglia and reduced LPS-induced inflammatory signaling through pathways involving NF-κB and c-Jun. Following experimental SCI, OEC-derived EV treatment reduced proinflammatory macrophage/microglial responses, increased repair-associated markers, preserved neurons and axons, and improved functional recovery [140].

OEC-EV activity is likewise producer-state dependent. EVs released from LPS-preconditioned OECs differed from those released under baseline conditions and produced stronger anti-inflammatory effects on microglia. The altered activity was associated with enrichment of miR-1224 and regulation of a CD44/SLC7A11 pathway [141]. Although this remains preclinical and the traditional M1/M2 terminology used in the study does not capture the full diversity of microglial states after SCI, the experiment provides a useful proof of principle. Conditioning the parent glial cell can deliberately change the biological activity of its EV product.

## 8. Replace–Reprogram–Relay: Matching the Intervention to the Pathology

Across these examples, the framework is best used as a decision map rather than an efficacy hierarchy. Replace and Reprogram specify biological requirements: replacement of a missing or intrinsically defective cellular population or function, or modification of surviving recipient cells without population replacement. Relay specifies the associated spatial and temporal delivery requirement when applicable. These requirements can coexist within the same disease and may therefore favor combination strategies. The condition-specific applications of this framework, including the mechanistically inferred roles of cell- and EV-based therapies and their corresponding levels of translational evidence, are summarized in Table 2.

Replace. Replacement is most compelling when pathology reflects loss or intrinsic dysfunction of a critical cell population, or loss of a structural function that only cells can provide. CSF1R-associated microgliopathy is the clearest microglial example, while severe failure of the myelin-forming compartment provides a parallel rationale for OPC transplantation [124,125,126,127]. Molecular modulation may improve the surrounding environment but cannot by itself restore a missing cellular compartment.

Reprogram. In acute SCI, stroke, chronic MS lesions, and other inflammatory settings, relevant cells may remain present but function maladaptively. The goal is to restore or redirect their behavior rather than increase cell number. EVs are one possible platform for addressing this Reprogram requirement because they can deliver coordinated signals to several recipient populations, including interacting immune, astrocytic, metabolic, and progenitor-cell programs involved in remyelination [33,34,142]. These responses should be described as modulation or phenotypic change unless coordinated transcriptional reprogramming or persistence after treatment withdrawal has been demonstrated.

Relay. Diffuse neurodegeneration, multifocal inflammation, and widespread proteopathy place the exposure problem at the center of therapy. Engineered EVs can provide defined, repeatable molecular delivery, whereas engineered microglia may provide persistent local production that changes with pathology [54]. Relay specifies the required distribution of therapeutic activity and does not prescribe a particular product. Broad CNS biodistribution should never be assumed for EVs since intact-vesicle delivery, target-cell uptake, and target engagement require product-specific demonstration.

**Table 2:** Condition-specific comparison of glial cell and extracellular vesicle therapeutic strategies in CNS disease. The “highest model/translation level represented” records the most clinically proximal directly relevant setting discussed for each platform (human clinical, non-human primate in vivo, rodent in vivo, or in vitro). It does not rank methodological quality, certainty of evidence, or therapeutic efficacy. Phase I/II safety or feasibility studies are not treated as efficacy evidence, and pathogenic EV observations are distinguished from therapeutic studies. References are representative examples and should be interpreted with the corresponding disease sections.

Condition	Dominant Repair Problem	Cell Approach	EV Approach	Mechanistically Inferred Role	Highest Model/Translation Level	References
CSF1R-associated microgliopathy	Genetically defective microglial population	Repopulates the defective microglial compartment with donor-derived myeloid cells	May provide molecular modulation but cannot directly replace the defective microglial population	Cell replacement favored when the cellular defect is causal	Cell: human clinical (small uncontrolled cohort) EV: no direct therapeutic evidence	[14,143]
Acute SCI	Secondary injury, vascular dysfunction, oxidative stress, and inflammatory signaling	Provides adaptive local support but requires invasive delivery	Can modulate inflammatory, vascular, and neuronal responses without cellular engraftment	EVs are attractive for early modulation of secondary injury while evidence remains largely preclinical	Cell: rodent in vivo EV: rodent in vivo	[63,73,144,145]
Chronic SCI	Cell loss, demyelination, axonal disconnection, and loss of trophic support	Can provide persistent cellular support, myelination, and potentially a permissive substrate for repair	Can modulate surviving cells but cannot directly replace lost cellular or structural components	Stage- and mechanism-dependent where combination strategies may be advantageous	Cell: human Phase I EV: no direct chronic-SCI therapeutic evidence discussed	[71,146]
Ischemic stroke	Injured but potentially salvageable peri-infarct tissue, vascular dysfunction, inflammation, and impaired plasticity	Provides trophic and immunomodulatory support and, in selected models, donor-derived glial replacement or remyelination.	Microglial EVs can modulate immune signaling, oligodendrogenesis, white-matter repair, and neurogenesis; effects are producer-state dependent	Distributed paracrine modulation where producer-cell state is critical	Cell: rodent in vivo EV: rodent in vivo	[75,76,77,78,79,80,81,82,83,84]
Progressive MS	Chronic glial dysfunction, impaired oligodendroglial repair capacity, and remyelination failure	May restore depleted or functionally impaired regenerative populations but still experimental	Can modify microglial, astrocytic, and OPC responses and influence remyelination	Mechanism- and stage-dependent; replacement remains experimental in MS	Cell: NHP in vivo (OPC transplantation) EV: rodent in vivo	[33,34,95,102]
Alzheimer’s disease	Proteinopathy together with altered microglial function and chronic neuroinflammation	Microglial engineering or donor-myeloid niche repopulation may restore selected microglial functions	Microglial EVs propagate Aβ associated synaptic dysfunction whereas microglia-derived macrosomes reduce plaque burden and improve cognition in AD-model mice	Potentially complementary though direct comparative evidence is lacking	Cell: rodent in vivo Pathogenic EV biology: rodent in vivo	[56,108,109]
Parkinson’s disease	α-Synuclein pathology, dopaminergic neuronal loss, and glial inflammation	Engineered or replacement microglial approaches remain highly experimental	Potential therapeutic delivery platform, while endogenous microglial EVs can propagate α-synuclein and inflammatory signaling	Requires rigorous control of producer-cell state and EV cargo	Cell: no direct therapeutic evidence discussed EV therapeutic: no direct therapeutic evidence discussed; pathogenic EV biology: human biospecimen + rodent in vivo	[110,147]
ALS	Diffuse motor-system degeneration with non-cell-autonomous glial toxicity and loss of trophic support	Can provide persistent local support where engineered neural/glial progenitors have entered early clinical testing	Schwann cell EVs administered clinically in a single-patient. Pathogenic astrocyte-derived EVs implicated in neuronal injury in vitro.	Potentially complementary though evidence levels remain asymmetric	Cell: human Phase I/IIa. Therapeutic glial EVs: human sin-gle-patient case report but efficacy not established. Pathogenic glial EV biology: in vitro.	[121,148,149]
Severe oligodendroglial loss/demyelination	Insufficient number or function of myelin-forming cells	Provides new oligodendrocytes capable of structural remyelination	May stimulate endogenous OPC recruitment/differentiation but cannot directly supply donor-derived myelin-forming cells	Cells required when structural cellular replacement is necessary	Cell: human Phase I/IIa (OPC transplantation in SCI; safety/feasibility); experimental remyelination: rodent in vivo. EV: rodent in vivo.	[15,33,127]

## 9. Translational Barriers: Making a Biologically Fair Comparison

### 9.1. Direct Parent Cell Versus EV Comparisons Remain a Critical Gap

Reviews often compare cell and EV therapies despite the scarcity of direct experiments under matched conditions. EVs may offer practical advantages, including lack of autonomous proliferation, redosing, and storage, but these attributes do not show that EVs reproduce parent-cell activity or that vesicular delivery is superior to simpler acellular alternatives. Mechanistic comparisons should include the parent cell, its EVs, EV-depleted conditioned medium, and, when appropriate, a soluble or non-vesicular delivery comparator.

A rigorous parent-cell/EV comparison should use the same donor and differentiation batch, define producer-cell state, control EV isolation, and match disease stage and route as closely as biology permits. EV-depleted conditioned medium helps separate vesicular from soluble secretome activity. Biodistribution, recipient-cell uptake, target engagement, mechanism, duration of effect, and long-term safety should accompany functional outcomes. Cargo- or pathway-loss-of-function experiments are especially valuable because they test whether vesicular transfer is required for an effect attributed to the parent cell.

Dose equivalence is a separate problem. Cell number cannot be converted directly into an EV particle dose: transplanted cells may persist and adapt their output, whereas each EV administration is a finite exposure. Comparisons should integrate physical dose descriptors with functional potency, target exposure over time, pharmacokinetics, and biodistribution rather than rely on arbitrary particle-to-cell conversions [150] (Table 3).

### 9.2. EV Identity Is Not EV Potency

MISEV2023 recommends operational terms such as “extracellular vesicle” or “small EV” when biogenesis has not been demonstrated, rather than assuming an exosomal origin [1]. EV preparations may contain multiple vesicle subpopulations together with non-vesicular particles, lipoproteins, soluble proteins, nucleic acids, and aggregates [1]. Particle size, concentration, and markers such as CD63, CD81, and CD9 characterize a preparation but do not establish therapeutic potency [1,5]. Paired proteomic studies show that neural-cell EV cargo is selectively represented relative to the producer cell and differs across lineages [151]. HiPSC-derived neuronal, astrocytic, microglial, and oligodendroglial EVs likewise retain parent-cell overlap while showing lineage-specific functional biases and enrichment of selected disease-associated proteins [152]. Producer-cell identity alone does not predict biological activity. Potency should be measured with a quantitative assay linked to the intended mechanism [5]. For example, a remyelinating EV should reproducibly enhance OPC differentiation or myelination, whereas a neuroprotective EV should protect the relevant neural population under a defined disease-relevant stress.

### 9.3. Manufacturing and Comparability

The acellular nature of EVs simplifies some aspects of product handling as isolated vesicles cannot proliferate or change lineage after administration, and repeated dosing may be easier to implement. Scale-up nevertheless remains technically demanding. Yield and composition vary with cell state, culture conditions, harvest interval, and purification. Tangential-flow filtration, size-exclusion chromatography, and combined workflows are being adapted to clinical-scale production, but recovery is useful only if the process preserves biological potency [3,153].

Cell-based products raise a different set of comparability questions, including post-thaw viability, differentiation state, genomic stability, proliferative potential, migration, phenotype after engraftment, and batch-to-batch consistency. Because a living graft can continue to change in vivo, release testing can define the administered product more precisely than the final biological state that develops after engraftment.

### 9.4. Biodistribution and Pharmacology

Neither platform has yet achieved reliable and predictable biodistribution within the CNS. Following systemic administration, many EV preparations are rapidly cleared into the liver, spleen, and other compartments of the mononuclear phagocyte system. CNS exposure depends on route, source, dose, formulation, and engineering rather than being an intrinsic property of EVs [154]. Fluorescent membrane dyes can persist after vesicle degradation or label non-EV material, so tissue fluorescence alone does not establish intact-vesicle delivery. Demonstrating CNS biodistribution should ideally include orthogonal evidence of intact particle delivery, recipient-cell uptake, cargo transfer, and target engagement.

The EV administration route should be selected according to required tissue exposure, pharmacokinetics, and demonstrated biological effect. Intravenous administration is clinically accessible, but systemically delivered EVs commonly undergo substantial uptake by peripheral organs, particularly the liver and spleen, which can limit CNS exposure [154]. Intranasal delivery has produced encouraging brain distribution in rodents, yet a non-human-primate pharmacokinetic study found minimal detectable exposure for the tested preparation compared with intravenous delivery [16,155]. Intrathecal or intraventricular administration provides direct CSF access but does not ensure efficient or uniform penetration into deeper brain or spinal-cord parenchyma [7]. Route selection should therefore be based on quantitative biodistribution and target-engagement data for the specific EV product and indication.

### 9.5. Safety and Clinical Development

EVs eliminate some graft-specific hazards but retain biological risk. They cannot form an expanding cellular mass, yet they can carry inflammatory, thrombogenic, immunogenic, oncogenic, or proteopathic material, and off-target uptake may produce systemic effects. Endogenous microglial EV pathways in AD and PD provide direct examples of vesicle-mediated disease propagation [106,107,108,109,110,111,113,147]. Cell therapies instead raise concerns about overgrowth, ectopic engraftment, immune rejection, inappropriate differentiation, conditioning toxicity, and effects that may be difficult to reverse. Safety assessment should be tailored to the liabilities of each product rather than assuming that an acellular product is intrinsically safe.

The clinical evidence remains asymmetric. Glial or glial-lineage cell approaches have human safety or feasibility data in ALS and SCI [71,121,126,130], and CSF1R-associated microgliopathy provides small-cohort human evidence consistent with altered disease trajectory after replacement of an abnormal myeloid compartment [14]. Therapeutic glial EV studies remain predominantly preclinical. A single-patient ALS report described intravenous allogeneic human Schwann cell-derived EV administration without infusion-related adverse events, although efficacy remains to be determined [149]. EV products from non-glial sources are entering clinical development, but the cited 2026 reviews report no EV therapeutic with regulatory marketing approval as of their respective literature cutoffs [3,6,7,8,156]. Table 2 reports the highest model/translation level represented for each platform without implying equivalent evidentiary strength or efficacy.

Regulatory considerations for EV therapeutics vary across jurisdictions and depend on product characteristics and intended use. In the United States, the FDA generally regulates exosome products intended to treat disease as drugs and biological products subject to applicable approval requirements. Regardless of the regulatory pathway, development should integrate source characterization, manufacturing and process controls, sterility assurance, impurity assessment, stability, potency, and comparability, alongside biodistribution studies and clinical pharmacology planning, from an early stage rather than only after preclinical efficacy has been demonstrated [7,157].

### 9.6. Preclinical Relevance and Reproducibility

Preclinical models often diverge from the intended clinical population. Many proof-of-concept studies use young animals, early treatment windows, and short follow-up, whereas neurodegeneration and chronic SCI involve aging, prolonged pathology, genetic heterogeneity, and systemic comorbidity. This mismatch is especially relevant to microglia because age, sex, genotype, region, prior inflammatory exposure, and ontogeny reshape microglial state [31,32,51,158].

Development programs should incorporate clinically relevant disease stages, both sexes where appropriate, aging when biologically material, randomized and blinded outcome assessment, and follow-up after treatment withdrawal. Human iPSC-derived multicellular systems, organotypic preparations, and ex vivo human tissue are most useful when they answer a mechanistic question not resolved by the animal model. Functional improvement is more persuasive when accompanied by target engagement, tissue-level mechanism, and a prespecified potency readout.

## 10. Experimental Priorities

Matched parent-cell/EV studies should use the same donor and differentiation batch, define producer-cell phenotype, include EV-depleted conditioned medium, apply orthogonal EV characterization, and incorporate mechanism-linked loss-of-function experiments. A relevant soluble or non-vesicular comparator should also be included when the claimed advantage is molecular delivery rather than cell replacement. Target engagement and duration of effect are more informative than forcing equivalence between cell number and particle number.

Producer-cell state should be linked directly to EV cargo and recipient-cell response. Targeted or single-cell profiling of producer cells combined with EV proteomics, lipidomics, RNA profiling, and functional assays can identify programs associated with protective versus deleterious vesicles. Microglia are particularly suitable because state transitions alter inflammatory signaling, lipid processing, phagocytosis, and remyelination-related pathways.

EV dose requires a pharmacological definition. Particle concentration is a descriptor, not a potency assay. Studies should quantify route-dependent exposure, clearance, intact-vesicle delivery where feasible, recipient-cell uptake, cargo transfer, target engagement, and functional potency across doses. A mechanism-linked potency assay should then support product release and comparability as manufacturing changes.

Engineered cell therapies require parallel development of controllability. Safety switches, inducible expression systems, immune engineering, and constraints on proliferative capacity may be needed as engraftment improves. For microglial replacement, donor-cell percentage alone is insufficient since ontogeny, state fidelity, spatial distribution, and long-term function also require measurement [43,46,47,48,51].

Duration of benefit should be measured explicitly. A transient response that requires repeated EV dosing and a persistent graft-mediated effect may look similar at an early time point but imply different mechanisms, risks, and treatment burdens. Chronic CNS disease requires follow-up long enough to distinguish acute signaling from sustained repair. Distinguishing durable recipient-cell state change from lasting tissue preservation requires repeated cell-specific molecular and functional measurements after EV withdrawal, with assessment of residual cargo activity and appropriate untreated and repeat-dose controls.

## 11. Limitations of the Review

This focused narrative review was not based on a prospectively registered systematic search, formal risk-of-bias assessment, or quantitative meta-analysis. Study selection therefore reflects mechanistic relevance and translational context rather than exhaustive retrieval, and the condition-specific assignments should not be interpreted as formal evidence grading. The literature is heterogeneous in species, disease stage, cell or EV product definition, route, dose metric, and follow-up, while matched parent-cell/EV studies remain uncommon. Importantly, most of the literature compared here was not generated specifically to answer cell-versus-EV questions, so the framework necessarily interprets evidence produced in asymmetric experimental programs. Microglia were used as the principal organizing lineage because both replacement and EV approaches are comparatively well developed. However, the framework may not generalize uniformly to astrocytes, oligodendroglial cells, Schwann cells, or other glial populations. Additional uncertainties include repeated EV dosing, immunogenicity, complement and coagulation responses, long-term exposure–response relationships, and performance in aged, comorbid, and human-relevant models. The framework should be read as a mechanistic structure for generating and testing therapeutic hypotheses, rather than as a quantitative efficacy comparison or recommendation for clinical use.

## 12. Conclusions

Glial cell and EV therapies address different therapeutic requirements. Cell-based approaches are most defensible when benefit depends on a function that must persist in vivo, including replacement of a genetically abnormal population, phagocytic surveillance, sustained local support, or generation of new myelin. EVs are plausible when relevant cells remain present and can be modified by a defined molecular exposure, especially when repeat dosing or distributed delivery is required. However, product-specific biodistribution, target engagement, and potency must still be demonstrated.

Microglia make this distinction unusually tractable because the same lineage can be replaced, engineered to respond to pathology, or used as a source of reparative or pathogenic vesicles. Producer-cell state, mechanism-linked potency, and therapeutic exposure are central variables in EV development and should be incorporated into primary product characterization.

The critical experiments are those that define the missing biological function and test competing ways to supply it. Matched studies of parent cells, their EVs, appropriate non-vesicular controls, target exposure over time, and duration of effect should establish the dominant biological mode (Replace or Reprogram, or both when justified) and the spatial/temporal delivery requirement (Relay, when applicable).

## Figures and Tables

**Table 1:** Distinct therapeutic properties of glia and their extracellular vesicles.

Therapeutic Property	Glial Cells	Glial EVs	Biological Implication	References
Environmental sensing after administration	Continuous	Absent	Important when lesion biology changes over time	[9,10]
Dynamic transcriptional response	Yes	No	Cells can alter therapeutic output after engraftment	[9,10]
Active migration toward pathology	Possible and cell-state dependent	No active migration; passive/engineered distribution	Active migration is a cellular property	[10,11,12]
Phagocytosis/debris clearance	Yes, for microglia/macrophage-lineage cells	No	Cannot be reproduced by an EV dose	[9,13]
Replacement of a defective cell compartment	Yes	No	Favors cells when the endogenous population is intrinsically abnormal	[10,14]
Structural myelination	Possible with OPC/oligodendroglial products	No	A cellular product supplies donor-derived myelin-forming cells	[15]
Sustained local trophic support	Potentially, months to years	Finite exposure where sustained effects require redosing or demonstrated downstream persistence	Persistent recipient-cell effects must be distinguished from continuous trophic delivery	[10,14,16]
Repeat dosing	Difficult and route dependent	Generally feasible	Practical advantage for EVs	[16,17]
Reversibility after administration	Limited after stable engraftment	Dosing can be stopped though downstream effects may persist	Relevant when dose control is important	[1,16]
Autonomous proliferation	Possible, source dependent	Absent	EVs avoid graft-overgrowth risk but retain cargo-related risk	[10,18]
Product heterogeneity	Cell-state and batch heterogeneity	Particle, cargo, and producer-state heterogeneity	Both require mechanism-linked quality control	[1,5,18]
Manufacturing challenge	Viability, phenotype, genomic stability, cryopreservation	Yield, purification, formulation, stability, potency in addition to cell measures of producer cell	Neither platform is intrinsically simple though EV manufacturing adds an extra layer of complexity	[1,5,18,19]

**Table 3:** Key experimental criteria for rigorous parent-cell versus EV comparisons.

Element	Why It Matters
Same donor, differentiation, and culture conditions	Removes source-related biological differences
Defined producer-cell state	Links EV activity to a measurable cellular phenotype
EV-depleted conditioned medium	Separates vesicular effects from soluble secretome activity
Orthogonal EV characterization	Reduces attribution of activity to contaminants or non-EV particles
Multiple dose metrics plus potency	Avoids treating particle number as a complete therapeutic dose
Matched disease stage and route, where feasible	Prevents timing and delivery from dominating the comparison
Biodistribution and recipient-cell uptake	Establishes whether target exposure is comparable
Mechanistic endpoints and target engagement	Moves interpretation beyond behavioral correlation
Cargo/pathway loss-of-function	Validates the functional contribution of selected cargo or pathways to EV-associated therapeutic effects without independently establishing vesicular transfer
Duration after treatment withdrawal	Tests persistence after exposure ends; sustained repair alone does not establish durable cellular reprogramming
Long-term safety	Captures platform-specific liabilities
Age, sex, genotype, and disease stage	Tests efficacy in the biological context intended for treatment
Independent replication and human-relevant models	Determines whether efficacy survives changes in species, laboratory, chronicity, and disease complexity
Exposure-normalized comparison	Cell number and EV particle number are not commensurate; compare functional exposure and target engagement over time
Relevant non-vesicular comparator	Tests whether vesicle packaging adds value beyond soluble secretome components or another molecular delivery platform

## Data Availability

No new data were created or analyzed in this study. Data sharing is not applicable to this article.
